# Power-Hop: A Pervasive Observation for Real Complex Networks

**DOI:** 10.1371/journal.pone.0151027

**Published:** 2016-03-14

**Authors:** Evangelos Papalexakis, Bryan Hooi, Konstantinos Pelechrinis, Christos Faloutsos

**Affiliations:** 1 Department of Computer Science, Carnegie Mellon University, Pittsburgh, PA, United States of America; 2 Machine Learning Department, Carnegie Mellon University, Pittsburgh, PA, United States of America; 3 School of Information Sciences, University of Pittsburgh, Pittsburgh, PA, United States of America; Inserm U869, FRANCE

## Abstract

Complex networks have been shown to exhibit universal properties, with one of the most consistent patterns being the scale-free degree distribution, but are there regularities obeyed by the *r*-hop neighborhood in real networks? We answer this question by identifying another power-law pattern that describes the relationship between the fractions of node pairs *C*(*r*) within *r* hops and the hop count *r*. This scale-free distribution is pervasive and describes a large variety of networks, ranging from social and urban to technological and biological networks. In particular, inspired by the definition of the fractal correlation dimension *D*_2_ on a point-set, we consider the hop-count *r* to be the underlying distance metric between two vertices of the network, and we examine the scaling of *C*(*r*) with *r*. We find that this relationship follows a power-law in real networks within the range 2 ≤ *r* ≤ *d*, where *d* is the effective diameter of the network, that is, the 90-th percentile distance. We term this relationship as power-hop and the corresponding power-law exponent as power-hop exponent *h*. We provide theoretical justification for this pattern under successful existing network models, while we analyze a large set of real and synthetic network datasets and we show the pervasiveness of the power-hop.

## Introduction

During the last decade a wealth of studies have identified an impressive set of universal network properties. Scale-free degree distributions [1–4], the presence of a giant component [5] and small average shortest paths coexisting with high clustering [6–9] are just some of them. The presence of scale-free distributions have attracted ideas from fractal theory and self-similarity [10] in the analysis of complex networks. An object is said to be self-similar if it appears the same in any length scales observed. For example, with *M* and *ρ* being the parameters of a self-similar object (e.g., mass and characteristic length respectively), the latter can be described through a power-law such as: *M* = *ρ*^*δ*^. Hence, fractal theory can quantify the dimensionality structure of such complex geometric objects beyond pure topological aspects based on similar scaling laws, since the exponent *δ* is the dimension of the scaling law. Furthermore, in contrast to topological dimensions, the fractal dimensions can take non-integer values allowing us to describe in greater detail the *space* that the object of interest fills [10]. In the case of complex networks these various dimensions carry information about many interesting underlying properties such as information diffusion and percolation [11–14]. While for networks embedded in a metric space the definitions can be applied almost unchanged [15, 16] this is not the case for the majority of the complex networks we study.

Our work is inspired by the definition of the fractal correlation dimension *D*_2_ on a cloud of points S. In particular, with *C*(*r*) being the fraction of pairs of points from S that have distance smaller or equal to *r*, S behaves like a fractal with intrinsic fractal dimension *D*_2_ in the range of scales *r*_1_ to *r*_2_ iff:

$$\begin{array}{c} C\left(r\right)\propto r^{D_{2}}\;\;\;\;\;\;r1\leq r\leq r2 \end{array}$$ (1)

An infinitely complicated set S would exhibit the above scaling over all the possible ranges of *r*. However, real objects are finite and hence, Eq (1) holds only over a specific range of scales. For example, a cloud of points uniformly distributed in the unit square, has intrinsic dimension *D*_2_ = 2, for the range of scales [*r*_*min*_, 1], where *r*_*min*_ is the smallest distance among the pairs of S.

In the case of a complex network the set S is simply the set of vertices V. Eq (1) can be applied unchanged for networks embedded in a metric space where any of the distance metrics of the space (e.g., Euclidean distance) can be utilized. Motivated by definition (1) we explore the following power-hop conjecture, which avoids the limitation to only spatially embedded networks. With *C*(*r*) being the fraction of pairs of nodes within hop-count *r* and *d* the 90-th percentile diameter the power-hop conjecture states:

**Conjecture 1 (power-hop conjecture)** *Given a network* G=(V,E), *C*(*r*) *follows a power-law relationship with r*, *in the range* [2, *d*], *i.e*., *C*(*r*) ∝ *r*^*h*^, 2 ≤ *r* ≤ *d*.

The above conjecture essentially implies that the plot of *C*(*r*) versus *r* in log-log scale will be a straight line with slope equal to the power-hop exponent *h*. Since, real networks are finite objects we expect the above scaling to hold only over a specific range—similar to the scale-free degree distribution that holds at the tail of the distribution in the majority of the cases [5]. In particular, we conjecture that this range begins at *r* = 2 and up to *r* = *d*. To reiterate, *d* is the effective diameter, that is, the distance within which 90% of the node pairs are. The contribution of this study is twofold. First, the empirical analysis of a large and diverse collection of network datasets (both real and synthetic) supports the power-hop conjecture. In particular, our results showcase the pervasiveness of this pattern. Of course, different types of networks exhibit different power-hop exponent. Second, we theoretically prove that under the successful Kronecker network model [17] this pattern is justified and preserved. We would like to note here that Faloutsos *et al*. [4] have shown that, for the Internet topology at both the autonomous system and router level, there is a power-law relationship between the hop distance *r* and the fraction of nodes within distance *r*. In our work we provide strong empirical evidence that this is also true in a diverse set of real networks (not only the Internet) within specific scales, supporting further the above conjecture.

## Materials and Analysis

**Materials:** For our analysis we use a large collection of publicly available network datasets. Table 1 presents basic meta-data information for the networks, while Table 2 provides some statistics about these datasets. For each of the networks we calculate the all-pairs shortest paths and compute *C*(*r*) as a function of the hop-count *r*. We then provide a linear fit log *C*(*r*) = *α* + *β* ⋅ log *r*, where essentially *β* = *h*.

**Table 1:** Network dataset meta-information. Basic information about the relationships that the real network datasets used for our power-hop analysis represent. For every network we define its nodes and edges, as well as its type (i.e., directed vs undirected). For the collaboration networks, HEP-TH captures the collaborations between high energy theoretical physicist, while the GR-QC captures the collaborations between physicist working on general relativity and quantum cosmology. Furthermore, the urban networks capture consecutive visitations by Foursquare users to venues in New York City and San Francisco respectively.

	Network Dataset	Nodes	Edges	Type
Technological	Power grid	Power stations	Physical connections	Undirected
	Internet AS	Autonomous Systems	Physical connections	Undirected
	Web	Web pages	Hyperlinks	Directed
Bio/Urban	Yeast Protein Interactions	Proteins	Protein interactions	Undirected
	Foursquare-NYC Urban	Urban venues	Consecutive visitations	Directed
	Foursquare-SF Urban	Urban venues	Consecutive visitations	Directed
Social	Gowalla	People	Friendships	Undirected
	Collaboration-HEP-TH	Scientists	Collaboration	Undirected
	Collaboration-GR-QC	Scientists	Collaboration	Undirected
	Facebook	People	Friendships	Undirected

**Table 2:** Pervasiveness of the power-hop scaling. Basic information about the different real network datasets used for our power-hop analysis. For every network we also provide the calculated *h* and the corresponding *R*^2^ of the fit, as well as the effective diameter *d*.

	Network Type	Source	|V|	|E|	*h*	*R*^2^	*d*
Technological	Power grid	[6]	4,941	6,594	2.48	0.99	27
	Internet AS	[18]	6,474	13,895	2.67	0.97	5
	Web	[19]	281,903	2,312,497	2.1	0.98	10
Bio/Urban	Yeast Protein Interactions	[20]	1,871	2,277	3.01	0.99	9
	Foursquare-NYC Urban	S1 Text	25,295	84,646	3.31	0.99	8
	Foursquare-SF Urban	S1 Text	7,944	27,629	3.16	0.99	7
Social	Gowalla	[21]	196,591	950,327	4.00	0.97	6
	Collaboration-HEP-TH	[22]	9,877	25,998	3.8	0.98	8
	Collaboration-GR-QC	[18]	5,242	14,496	3.51	0.99	8
	Facebook	[23]	63,731	817,035	3.9	0.96	6

We also build synthetic network datasets using two well-studied network formation models, namely, the Barabási-Albert (BA) preferential attachment [1] and the stochastic Kronecker graph model [17] that has been shown to be able to reconstruct many of the (static and temporal) patterns that real network datasets exhibit. The BA model has two parameters that we can tune: the number of edges *m* that every new node generates in the network and the power *w* of the preferential attachment (e.g., for *w* = 1 we have a linear preferential attachment). In brief, with BA model at every iteration a new node *i* is generated. This node further generates *m* new edges whose one end is attached to *i* while the other end is randomly attached to an already existing nodes *j* in the network, with a probability proportional to ${\text{deg}}_{j}^{w}$, where deg_*j*_ is *j*’s degree. The highest the degree of a node the more chances it has to acquire more edges; “the rich gets richer”. Furthermore, a larger value for *w* pronounces this phenomenon leading to the generation of the “hub” nodes that attract the majority of the edges. In our experiments, we used the following sets of parameters: (*m*, *w*) = {(1, 0.5), (1, 0.65), (1, 0.9), (1, 1), (2, 0.5), (2, 0.65), (2, 0.9), (2, 1)}. The seed network for the BA model consists of two connected nodes. For every parameter setting we generated 100 topologies with 10,000 nodes and calculated *h*, *d* and the *R*^2^ of the linear fit.

The stochastic Kronecker graph model accepts as input a probability matrix $P_{1}\in \Pi^{N_{1}\times N_{1}}$ and an integer value *k*. $P_{1}$ describes a matrix that represents a small initiator network *G*, with the entry *p*_*ij*_ describing the probability of the edge (*i*, *j*). Using $P_{1}$ we compute its *k*^*th*^ Kronecker power (see S2 Text) and obtain matrix $P_{k}=P_{1}^{⊗k}$. Then for every pair of nodes (*u*, *v*) we include an edge with probability *p*_*k*_*u*, *v*__ and the corresponding network is *G*^*k*^. In our experiments, we use *k* = 5 and *N*_1_ = 5, while *p*_*ij*_ = *γ*, ∀ *i*, *j*{1, …, *N*_1_} with *γ* ∈ [0.3, 0.5].

**Analysis:** Can we prove under realistic assumptions that networks will exhibit the behavior expected from the power-hop conjecture? Fast forwarding, the answer is yes and for our theoretical analysis we rely on the Kronecker model. The reason for using the latter, is that the Kronecker model (being a superset of the successful Recursive Matrix model [24]) has been shown to be able to match a number of static and time-evolving properties of networks [17]. Hence, with this model being a realistic assumption, we are interested in examining on whether it exhibits the power-hop scaling behavior observed in real network datasets. Furthermore, its mathematical tractability allows for analytical derivations. In particular, we have the following lemma, which we prove in the supplementary materials (see S3 Text):

**Lemma 1** *Let M be a binary m* × *m matrix that describes the seed network G*. *Then in G*^*K*^, *the number of pairs reachable in r hops is* $c_{r}^{K}$, *where c*_*r*_ *is the number of pairs reachable in r hops in G*.

Simply put, the above lemma says that Kronecker multiplications will retain the power-hop scaling. As a consequence of Lemma 1, if the initial network *G* has power-hop exponent *h*_1_, then after *K* Kronecker products/iterations, the resulting network will have a power-hop exponent *Kh*_1_. While Lemma 1 considers the simple case of binary seed matrix, the following Lemma considers the generic case of stochastic Kronecker model (see S4 Text for the proof)

**Lemma 2** *Let M be a seed matrix, and let the edges of G be generated based on the probabilities in M, and G*^*K*^ *generated based on M*^⊗*K*^. *Then in G*^*K*^, *as K* → ∞, *the expected number of pairs reachable in r hops approaches* $c_{r}^{K}$, *where c*_*r*_ *is the expected number of pairs reachable in r hops in G*^*K*^.

Before describing our experimental results in detail we would like to emphasize here on some practical details for estimating the power-hop exponent. In particular, the formal definition of *D*_2_ is the following:

$$\begin{array}{c} D_{2}=\lim_{r\to 0}\frac{\log C(r)}{\log r} \end{array}$$ (2)

Theoretically, this limit provides the slope of the straight-line dependence between log*C*(*r*) and log*r*. Nevertheless, given that any practical/real dataset is finite, the above scaling holds only over a small range for *r* and the correlation dimension is computed from the slope over this range [10]. This is actually the case in our network datasets as well and we are following the literature on the practical fractal dimensions [10] to compute the power-hop exponent.

## Results

Next we present the experimental results obtained from the real and synthetic datasets.

**Real network datasets:** We begin by analyzing various network datasets that represent different types of systems. In particular, we have analyzed technological (e.g., power grid, the Internet and the web-graph), social (e.g., friendship networks—Facebook, Gowalla—and co-authorship networks), urban (e.g., associations between locations based on human mobility patterns) and biological networks. Our results are presented in Table 2, where we also provide a pointer to the data sources. Note here that, for networks that are not connected, we analyze the largest-connected component.

**Fig 1:**
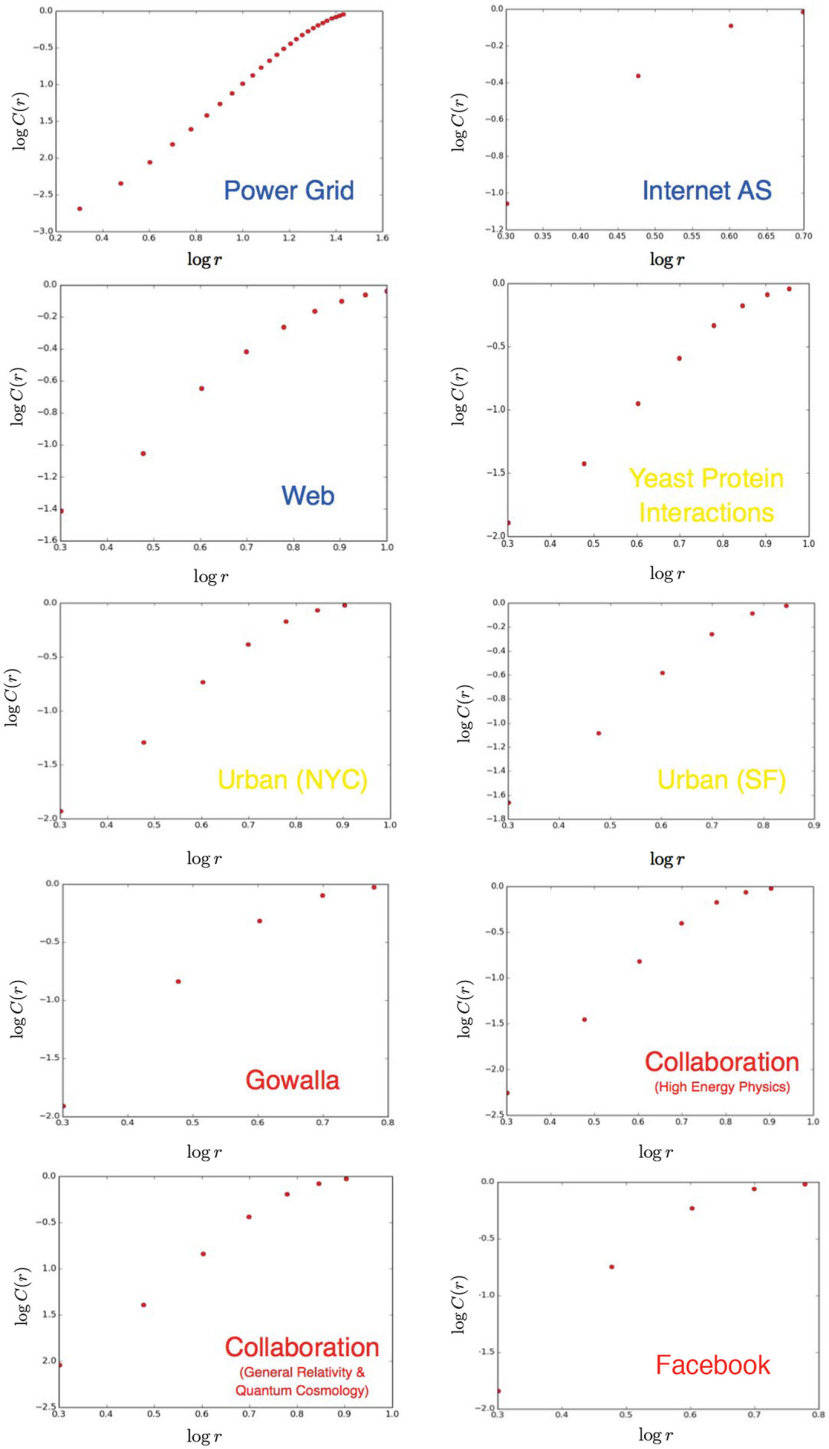
power-hop scaling for our network datasets. The power-hop conjecture appears to be true for all the network datasets we examined despite the fact that they capture completely different network dynamics.

As we can see the pattern is pervasive and the linear fit is very good for all of the cases (*R*^2^ > 0.96) even for those with networks with large effective diameter. Fig 1 depicts the corresponding dependencies between *C*(*r*) and *r* for all the networks in log-log scale. Only the range [2, *d*] is plotted for clarity.

**Fig 2:**
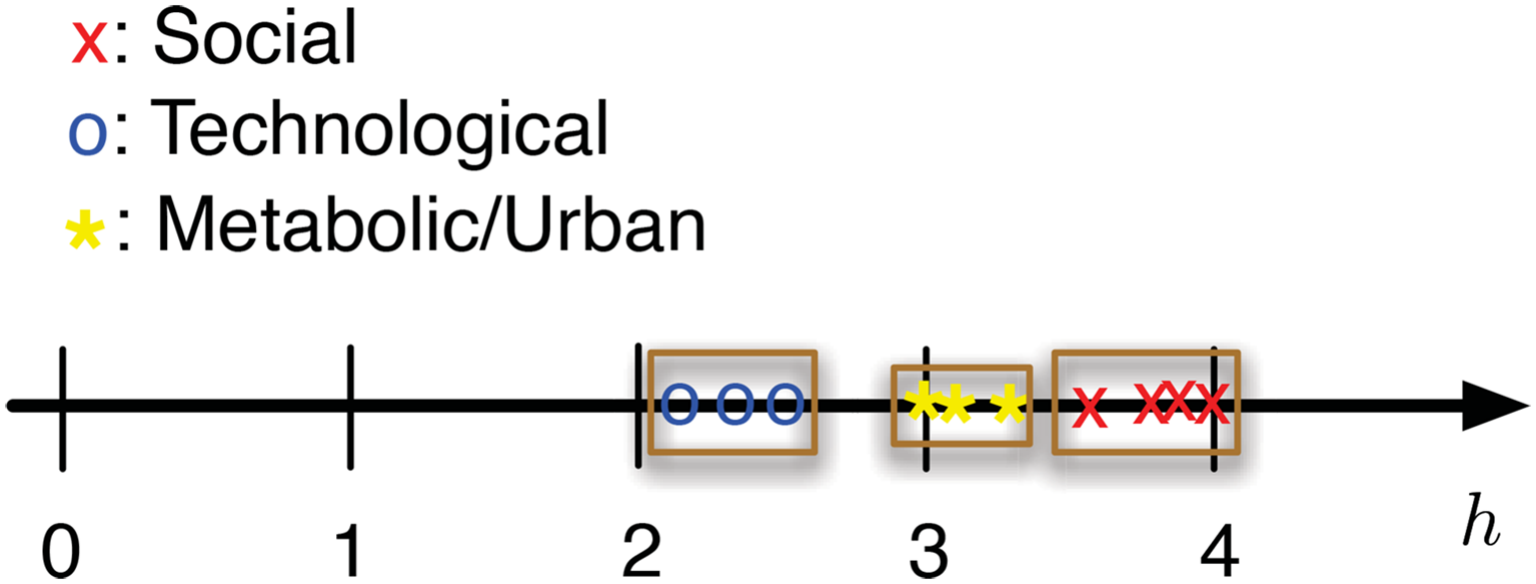
power-hop exponents of various types of networks. The power-hop exponent *h* of similar types of networks cluster well together. Interactions within a city have been described through biological metaphors and hence, it is logical that the exponents of our urban network datasets is close to that of the biological networks.

Finally, we would like to emphasize here that the power-hop exponent of networks of the same type are very similar and are nicely ordered on the real number line (see Fig 2). For example, social networks have a power-hop exponent roughly in the range 3.5–4, while technological networks have a much smaller exponent, that is, in the range 2–2.5. The only biological network in our datasets (a protein-protein interaction network) also exhibits a different exponent (*h* ≈ 3), while the urban networks examined exhibit exponents in the range 3–3.5. The latter, while capturing aggregated urban mobility patterns, have been created from and affected by the underlying social network layer. Furthermore, interactions within a city have been described through biological metaphors in both classical and recent studies [25–29]. Therefore, it might be logical that the power-hop exponent of our urban network datasets is close to that of both the biological and social networks. We would like here to emphasize on the fact that the groups depicted in Fig 2 are not clusters in the formal notion of unsupervised learning literature. The main point is that the power-hop exponent *h* of the networks of different types are ordered on the real line. In order to perform a robust cluster analysis one would need access to a much larger number of network datasets. Nevertheless, our results in this work show that *h* can potentially be used to classify/identify the type of a recorded network. Furthermore, given the consistency of the values for the power-hop exponent, network models that try to *explain* the link formation for different types of networks could potentially be evaluated on the basis of being able to reproduce this scaling as well.

**Fig 3:**
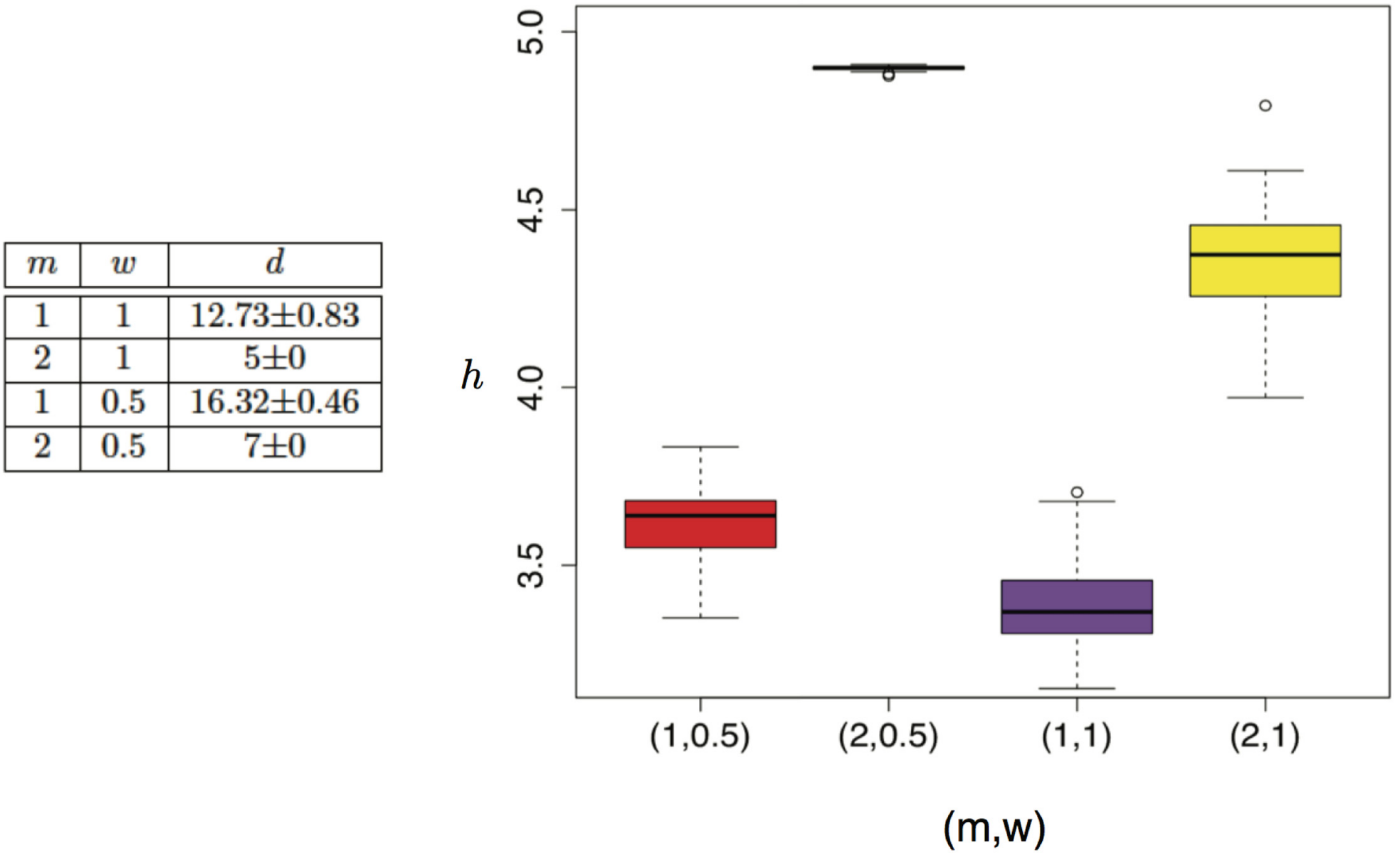
power-hop exponent *h* for two different types of the BA preferential attachment networks. Increasing the number of edges *m* that a new node generates leads to a decrease in the (effective) diameter since more edges can create shortcuts in the network with higher probability. Sub-linear attachment also leads to higher (effective) diameter, since the *hub* nodes do not attract the new edges as strongly as in the linear attachment (see table on the left). Reducing the number of edges while keeping the number of nodes and preferential attachment type constant generates less complex networks (networks with a smaller power-hop exponent). A similar result occurs if we increase the preferential attachment probability while keeping the number of nodes and edges constant.

**Synthetic network datasets—BA model:** Now we turn our attention to the synthetic datasets created using the BA network formation model. Fig 3 presents our results. In particular, we depict the box plots for the power-hop exponent *h* computed over all the networks generated with a given set of parameters. As per the power-hop conjecture, *h* is computed over the range [2, *d*].

As alluded to above, we examined networks generated with linear and sub-linear preferential attachment probabilities. We did not examine super-linear growth of the preferential attachment probability, since this leads to networks with extremely small diameter that do not allow us to obtain reliable statistical results due to the limited range between 2 and *d*. For each case we also considered two different scenarios, where each new node in the network generates one or two new edges. The effective diameter for each scenario is provided in the table at the left part of Fig 3 with the corresponding variance in parenthesis. Note here that, the effective diameter exhibits very small variance over the different network instances generated by the same model parameters. Our results for *h* are further depicted at the box plots in Fig 3. Note here that, the quality of the power-law fit was excellent for all cases (**R**^**2**^ **>** **0.98** in all networks). As we can see, if we focus on a specific value for the number of new edges added at each step (i.e., fixed *m*), linear (*w* = 1) preferential attachment provides networks with smaller power-hop exponent as compared to sub-linear preferential attachment (*w* ∈ {0.5, 0.65, 0.9}). In fact, we performed statistical tests (t test) for every pair of the model parameters and all rejected the null hypothesis at the significance level *α* = 0.05 (i.e., that the average power hop exponent *h* is the same for the two scenarios). Only exception is the comparison between (1, 0.5) and (1, 0.65) where our hypothesis test cannot reject the null at *α* = 0.05 (*p* − *value* = 0.33). Nevertheless, the dynamic range obtained for *h* when *m* = 1 is fairly small and so are the differences, especially for values of the preferential attachment *w* that are close to each other. Furthermore, for a given type of preferential attachment we observe that increasing the number of edges added by every new node, leads to an increase in the power-hop exponent of the complex network. We can draw some intuition behind this behavior, if we recall the connection between the power-hop exponent of our network and the fractal correlation dimension of a point-set. In general, higher dimensionality is associated with higher degree of complexity, and hence, a larger power-hop exponent can be deemed as a sign of a more complex network structure. In the case of the BA model, larger *w* leads to networks with well defined attractors, which further give raise to a well-defined structure (e.g., a few hubs and every other vertex is connected to these few central entities). A well-defined structure exhibits *regularity* and less complexity and hence, its dimensionality (which in our case is captured through *h*) is lower. Conversely, when *w* is small, there are not clear attractors that emerge, and the structure is less clearly defined, requiring in a sense more details/features to describe the system in detail (i.e., higher dimensionality). Similarly, larger *m* leads to more complex topologies, since for a given *w* there are more chances for a node to emerge as an attractor.

**Fig 4:**
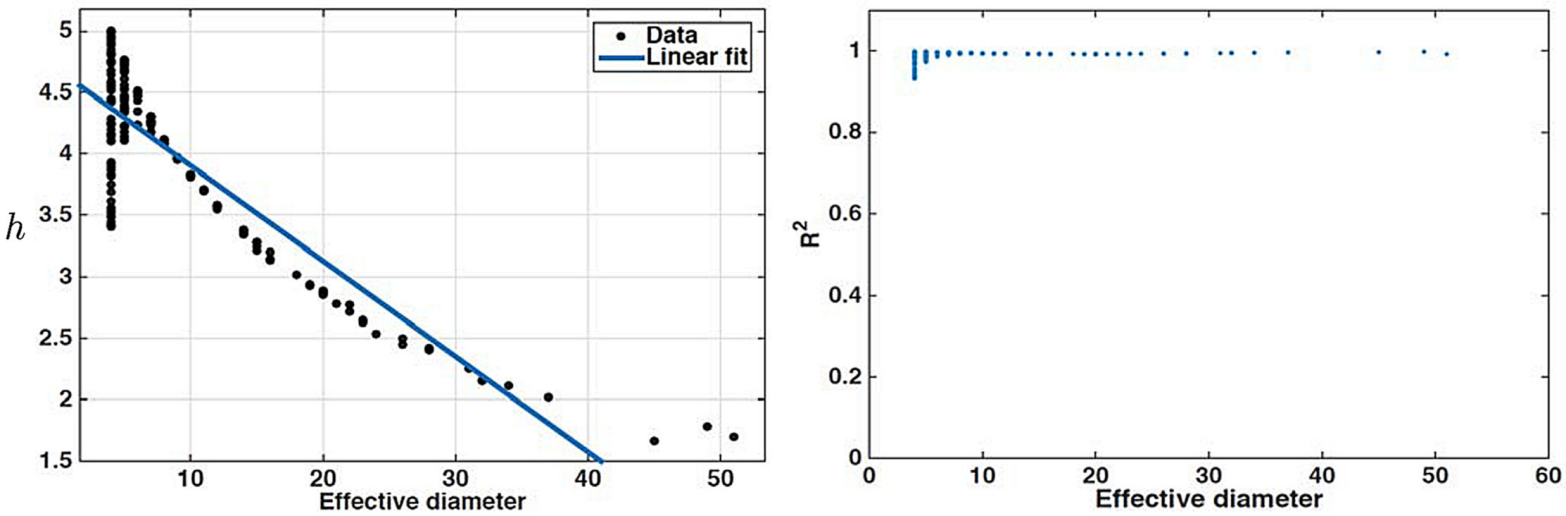
power-hop exponent for Kronecker networks. The networks generated with the Kronecker network model exhibit effective diameters that span a large range. The linear fit obtained for the *h* of every network exhibits large *R*^2^ values even for larger *δ*.

**Synthetic network datasets—Kronecker model:** Finally, we perform experiments using the stochastic Kronecker graph formation model. To reiterate, being a superset of the successful recursive matrix network model [24], the Kronecker model is one of the most successful network formation models that can recover many of the properties of real networks [17]. Hence, we are interested in examining whether Kronecker networks obey the power-hop conjecture. In particular, as alluded to above, we choose uniformly at random the value for *γ* in the range [0.3, 0.5]. Different values of *γ* will provide networks with different diameters (generally speaking larger *γ* provides networks with smaller diameter). As with the real network datasets and the synthetic datasets obtained from the BA model, the power-law fit Eq (1) is excellent again, with an **R**^**2**^ **>** **0.95** (see S5 Text). The left part of Fig 4 depicts a scatter plot of the fractal dimension for each obtained network and the corresponding effective diameter. As we can notice there is a clear decreasing trend, which is also in agreement with our results from the BA network model. In the same figure we plot a linear fit with slope −0.07 (p-value < 0.05) and *R*^2^ = 0.77. Furthermore, it is interesting to note that networks with smaller diameter exhibit a higher variance in the corresponding fractal dimension (somehow evident in the results from the real datasets as well). However, it is not clear whether this is an intrinsic phenomenon or just an artifact of the larger sample of networks with smaller diameter. Fig 4 (right part) presents a scatter plot of the *R*^2^ of the calculated fractal dimension as a function of the effective diameter. The reason for presenting these results stems from the fact that when *δ* is small, one can argue that it is *easier* to fit a line through the smaller number of points. Nevertheless, our results show that when the effective diameter is large, the *R*^2^ value is extremely high as well, further strengthening our observations for a universal network pattern. Finally, it is interesting to note that even the worst fit (which appears for the smallest effective diameter observed) is still fairly good (*R*^2^ = 0.9327).

**Fig 5:**
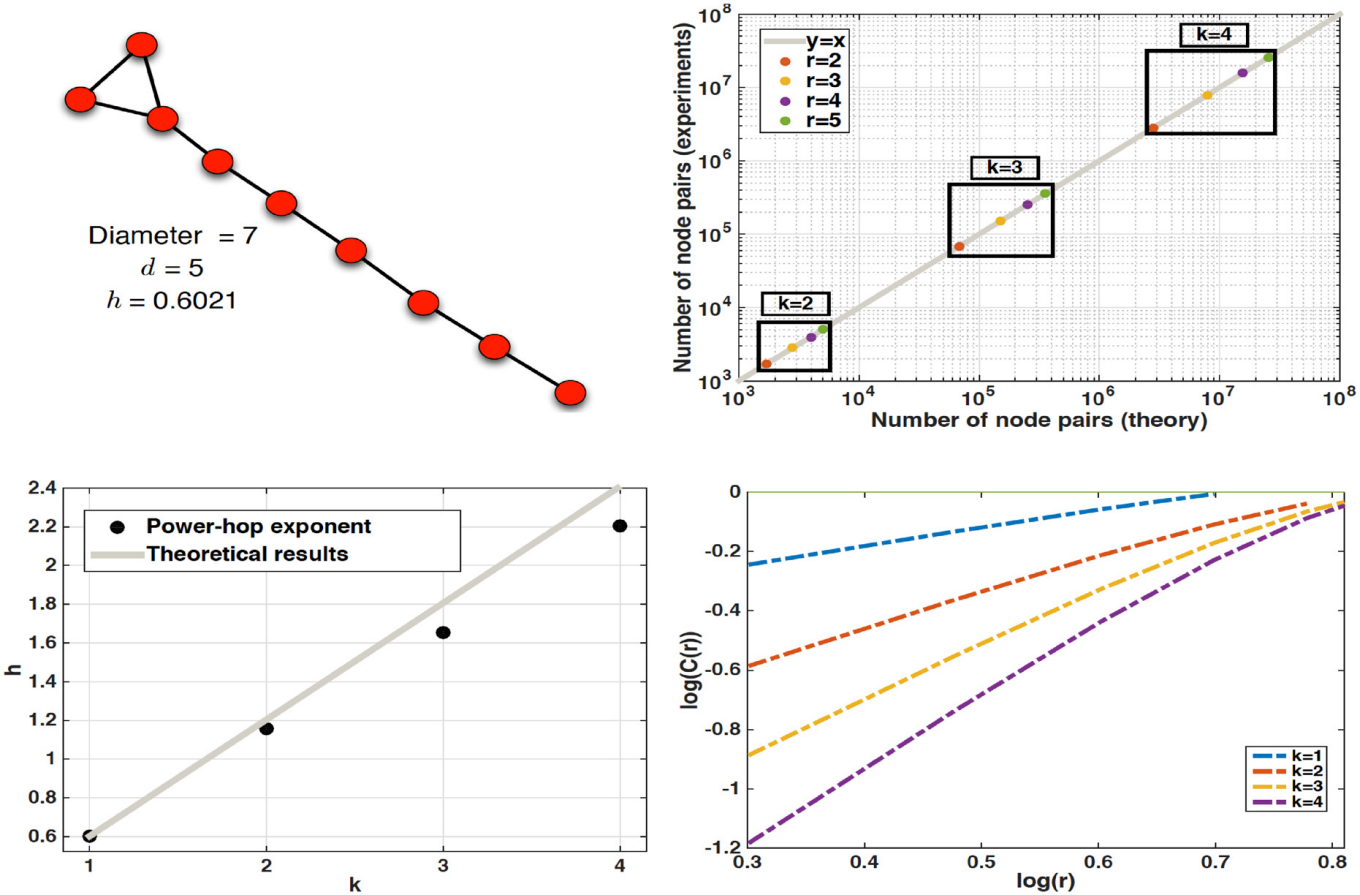
Numerical demonstration of Lemma 1. Using a seed network with large enough diameter to obtain statistically meaningful results (top left figure), we validate Lemma 1. As we can see at the top right figure the number of pair of nodes within distance *r* expected from Lemma 1 is equal to the one computed from the networks. Furthermore, the computed power-hop exponent is very close to the one expected from our lemma as well (bottom left figure). Finally, the actual scaling behavior for the networks after each Kronecker iteration are presented at the bottom right figure.

Finally, we experimentally demonstrate Lemma 1. We start with a seed matrix that describes the network depicted in the left part of Fig 5. This is essentially a network that exhibits a linear relationship between log*C*(*r*) and log*r* with a slope of *h*_1_ = 0.6021 and effective diameter *d* = 5. We then compute the *k*^*th*^ Kronecker power, *k* ∈ {2, 3, 4}, and experimentally obtain the number of pairs of nodes within distance *r* ∈ {1, …, *d*} for each network. The top-right part of Fig 5 presents this experimentally obtained number versus the theoretical one expected from Lemma 1. As we can see all points fall on the *y* = *x* line, which demonstrates the validity of Lemma 1 in this example. The bottom left part of Fig 5 presents the corresponding power-hop exponent *h* of each network obtained. As we can see again, the power-hop exponent expected as a consequence of Lemma 1 is very close to the one computed from the actual networks. The small difference between the theoretical and experimental values can be attributed to the fact that even though the *R*^2^ value of the linear fit for *h* is high, it is never perfect (i.e., 1). Finally, the bottom right part of Fig 5 depicts the actual scaling behavior of the networks after each Kronecker multiplication.

## Discussion

As aforementioned there is a volume of work in the literature that studies the dimensionality of networks (e.g., [11–15, 30–32]—with the list of course not being exhaustive). This body of literature studies the theoretical properties (e.g., phase transitions) and asymptotic behavior of the complex networks. However, despite the significant contribution of these studies, real networks are finite. Therefore, in our study we are more interested in the practical extensions of the existing literature, by analyzing and studying real-network datasets. Inspired by the fractal correlation dimension *D*_2_ defined over a point-set, we examine the scaling behavior of the fraction *C*(*r*) of node pairs within hop-count *r* and pose the power-hop conjecture (see Conjecture 1).

To summarize, the contributions of our work are as follows:

- **Pervasiveness:** all the real networks we studied support the power-hop conjecture, that is, they exhibit excellent fit for a power-law *C*(*r*) ∝ *r*^*h*^ (see Table 2)
- **Analysis:** we theoretically proved that one of the most realistic models, the Kronecker model, automatically leads to power law behavior of the power-hop conjecture, given mild initial conditions (see Lemma 1)

Furthermore, our empirical results (Fig 2) show that the value of the power-hop exponent is related to the type of network. While different networks exhibit different power-hop exponent, networks of the same type have similar exponents.

## Supporting Information

S1 Text Urban network dataset. (PDF)

S2 Text Kronecker product. (PDF)

S3 Text Proof of Lemma 1. (PDF)

S4 Text Proof of Lemma 1. (PDF)

S5 Text power-hop and Kronecker network model. (PDF)
