# Supplementary material for: Power-Hop: A Pervasive Observation for Real Complex Networks
Source: PLoS One. 2016 Mar 14;11(3):e0151027. doi: 10.1371/journal.pone.0151027 (PMC4790966; doi:10.1371/journal.pone.0151027)
Supplement: S1 Text — (PDF) [file pone.0151027.s001.pdf]

S1 Text. Urban network dataset. Our urban network dataset captures various aspects of the aggregated mobility patterns recorded through the Foursquare social network. In particular, using Foursquare's public venue API (`NextVenues` endpoint) we obtain for every Foursquare venue v, a set $\mathcal{V}_v$ of venues that users typically visit after v. The results are based on the number of users that have performed the transition $v \rightarrow u, u \in \mathcal{V}_v$. We have queried the whole Foursquare venue database and we have obtained a snapshot of the underlying network from March 3rd, 2015. We further filter only venues in New York City (NYC) and San Francisco (SF).

We would like to emphasize here that these datasets capture the interactions between the urban environment in the corresponding cities as recorded through social media users' check-ins. Of course, we acknowledge that not all of the underlying interactions are captured, since the API returns only up to 5 transitions (even though these are the dominating transitions observed), and furthermore for privacy reasons does not return any residence locations in set $\mathcal{V}_u$. However, it is a good representation of the underlying dynamics and most importantly our study is not expected to be affected by the missing data, since we observed almost identical trend in our results when calculating the `power-hop` exponent h over random samples of pairs nodes in our networks.
