# Supplementary material for: Power-Hop: A Pervasive Observation for Real Complex Networks
Source: PLoS One. 2016 Mar 14;11(3):e0151027. doi: 10.1371/journal.pone.0151027 (PMC4790966; doi:10.1371/journal.pone.0151027)
Supplement: S2 Text — (PDF) [file pone.0151027.s002.pdf]

S2 Text. Kronecker product. The Kronecker product, in contrast to the traditional matrix product, can be defined between matrices of any dimensions. In particular, let us consider matrix $\mathbf{X} \in \Pi^{m \times n}$ and matrix $\mathbf{Y} \in \Pi^{m' \times n'}$. Their Kronecker product $\mathbf{Z}$ is a $(m \cdot m') \times (n \cdot n')$ matrix, given by:

$$\mathbf{Z} = \mathbf{X} \otimes \mathbf{Y} =: \begin{pmatrix} x_{1,1}\mathbf{Y} & x_{1,2}\mathbf{Y} & \cdots & x_{1,n}\mathbf{Y} \\ x_{2,1}\mathbf{Y} & x_{2,2}\mathbf{Y} & \cdots & x_{2,n}\mathbf{Y} \\ \vdots & \vdots & \ddots & \vdots \\ x_{m,1}\mathbf{Y} & x_{m,2}\mathbf{Y} & \cdots & x_{m,n}\mathbf{Y} \end{pmatrix} \quad (1)$$
