# Supplementary material for: Power-Hop: A Pervasive Observation for Real Complex Networks
Source: PLoS One. 2016 Mar 14;11(3):e0151027. doi: 10.1371/journal.pone.0151027 (PMC4790966; doi:10.1371/journal.pone.0151027)
Supplement: S3 Text — (PDF) [file pone.0151027.s003.pdf]

S3 Text. Proof of Lemma 1. $M^{\otimes K}$ has m^K rows which can be identified with the m^K m-ary numbers of length K in order, labelling the rows from top to bottom. From the definition of the Kronecker product, there is an edge between node i, with m-ary number $i_1 i_2 \ldots i_K$, and node j, with m-ary number $j_1 j_2 \ldots j_K$, iff $M(i_k, j_k) = 1$ for all $k = 1, \ldots, K$. In other words, i and j are adjacent in G^K iff each m-ary digit of i and j are adjacent according to G. This implies that i and j are (mutually) reachable in r hops iff for each k, i_k and j_k are reachable in r hops according to G. For each k, c_r pairs of the form (i_k, j_k) are reachable in r hops (in G); therefore, c_r^K pairs are reachable in r hops (in G^K).
