# Supplementary material for: Power-Hop: A Pervasive Observation for Real Complex Networks
Source: PLoS One. 2016 Mar 14;11(3):e0151027. doi: 10.1371/journal.pone.0151027 (PMC4790966; doi:10.1371/journal.pone.0151027)
Supplement: S4 Text — (PDF) [file pone.0151027.s004.pdf]

S4 Text. Proof of Lemma 2. For any matrix M, let sum(M) denote the sum of its entries. The (i, j)th entry of $M^{\otimes K}$ is the probability of an edge existing between i and j in G^K. Consider a particular r hop path between i and j, which we denote $(z_0 = i, z_1, \ldots, z_r = j)$. The probability that G^K contains this path is $\prod_{k=1}^{r}(M^{\otimes K})_{z_{k-1},z_k}$. Thus, as $K \to \infty$ and using the approximation $\exp(-x) \to (1-x)$ as $x \to 0$, the probability that G^K contains at least one r hop path between i and j is:

$$
\begin{aligned}
&1 - \prod_{z_1,\ldots,z_{r-1}} (1 - \prod_{k=1}^{r}(M^{\otimes K})_{z_{k-1},z_k}) \\
&\to 1 - \prod_{z_1,\ldots,z_{r-1}} (\exp(-\prod_{k=1}^{r}(M^{\otimes K})_{z_{k-1},z_k})) \\
&= 1 - (\exp(-\sum_{z_1,\ldots,z_{r-1}} \prod_{k=1}^{r}(M^{\otimes K})_{z_{k-1},z_k})) \\
&\to \sum_{z_1,\ldots,z_{r-1}} \prod_{k=1}^{r}(M^{\otimes K})_{z_{k-1},z_k}) \\
&= ((M^{\otimes K})^r)_{i,j}
\end{aligned}
$$

Now, the expected number of pairs reachable in r hops in G^K is sum$((M^{\otimes K})^r)$. A known property of Kronecker products is that $(M^{\otimes K})^r = (M^r)^{\otimes K}$ (e.g. Corollary 4 in [1]). Hence,

$$
\begin{aligned}
\text{sum}(M^{\otimes K})^r &= \text{sum}((M^r)^{\otimes K}) \\
&= (\text{sum}(M^r))^K \\
&= (c_r)^K
\end{aligned}
$$

The second equality above follows from repeatedly applying the property that sum$(A \otimes B) =$ sum$(A) \cdot$ sum(B) for any matrices A, B, which can be shown by factorizing the sum of entries on the left hand side.

References

[1] H. Zhang and F. Ding (2013) On the Kronecker Products and Their Applications. Journal of Applied Mathematics vol. 2013, Article ID 296185, 8 pages.
