# Supplementary material for: Power-Hop: A Pervasive Observation for Real Complex Networks
Source: PLoS One. 2016 Mar 14;11(3):e0151027. doi: 10.1371/journal.pone.0151027 (PMC4790966; doi:10.1371/journal.pone.0151027)
Supplement: S5 Text — (PDF) [file pone.0151027.s005.pdf]

S5 Text. `power-hop` **and Kronecker network model.** Our experiments with networks generated by the Kronecker model seem to imply that the `power-hop` scaling can appear after a finite number of iterations (not a fixed number for all cases) even when the seed matrix does not satisfy the strict requirement of the power-hop property as per Lemmas 1 and 2. However, this is only a hypothesis that needs to be further theoretically proven. Identifying less strict requirements for the seed matrix can further generalize the theoretical results presented in this work.
